# Improving coronary heart disease self-management using mobile technologies (Text4Heart): a randomised controlled trial protocol

**DOI:** 10.1186/1745-6215-15-71

**Published:** 2014-03-04

**Authors:** Leila Pfaeffli Dale, Robyn Whittaker, Yannan Jiang, Ralph Stewart, Anna Rolleston, Ralph Maddison

**Affiliations:** 1 National Institute for Health Innovation, University of Auckland, 261 Morrin Rd, Auckland 1072, New Zealand; 2 Department of Cardiology, Auckland City Hospital, 2 Park Rd, Auckland 1023, New Zealand; 3 Department of Medicine, University of Auckland, 2 Park Rd, Auckland 1023, New Zealand

**Keywords:** Cardiovascular disease, Health behaviour, Lifestyle change, mHealth, Text messaging

## Abstract

**Background:**

Cardiac rehabilitation (CR) is a secondary prevention program that offers education and support to assist patients with coronary heart disease (CHD) make lifestyle changes. Despite the benefits of CR, attendance at centre-based sessions remains low. Mobile technology (mHealth) has potential to reach more patients by delivering CR directly to mobile phones, thus providing an alternative to centre-based CR. The aim of this trial is to evaluate if a mHealth comprehensive CR program can improve adherence to healthy lifestyle behaviours (for example, physically active, fruit and vegetable intake, not smoking, low alcohol consumption) over and above usual CR services in New Zealand adults diagnosed with CHD.

**Methods/design:**

A two-arm, parallel, randomised controlled trial will be conducted at two Auckland hospitals in New Zealand. One hundred twenty participants will be randomised to receive a 24-week evidence- and theory-based personalised text message program and access to a supporting website in addition to usual CR care or usual CR care alone (control). The primary outcome is the proportion of participants adhering to healthy behaviours at 6 months, measured using a composite health behaviour score. Secondary outcomes include overall cardiovascular disease risk, body composition, illness perceptions, self-efficacy, hospital anxiety/depression and medication adherence.

**Discussion:**

This study is one of the first to examine an mHealth-delivered comprehensive CR program. Strengths of the trial include quality research design and in-depth description of the intervention to aid replication. If effective, the trial has potential to augment standard CR practices and to be used as a model for other disease prevention or self-management programs.

**Trial registry:**

Australian New Zealand Clinical Trials Registry: ACTRN12613000901707

## Background

Unhealthy lifestyle behaviours, including smoking, physical inactivity, hazardous alcohol consumption and low intake of fruit and vegetables have been shown to contribute to the development of coronary heart disease (CHD) [1,2], which remains a leading cause of death worldwide [3]. Lifestyle modifications are encouraged for secondary prevention of CHD. Cardiac rehabilitation (CR) is a cost-effective program of education and support designed to assist patients make healthy lifestyle changes and adhere to clinical guidelines, and it is associated with reduced mortality and hospitalisations [4-7]. Recommended lifestyle changes can include starting and maintaining regular exercise, eating a heart-healthy diet, stopping smoking, adhering to prescribed medication regimens and attending medical appointments [8-10].

Inviting patients hospitalised for CHD to attend centre-based CR is usual care in most developed countries, including New Zealand, yet attendance rates remain low [11-13]. Barriers to attending CR include limited transportation and parking availability, inconvenient scheduling of CR sessions, language-related communication difficulties and employment or family demands [14-16]. Alternative delivery methods of CR are required to better meet patients’ needs.

Delivering CR using mobile technologies, known as mHealth, has the potential to overcome such barriers, as programs can be personalised and delivered anywhere and at any time. mHealth interventions have successfully been shown to have a positive effect on health behaviours, such as smoking cessation and improvement in disease self-management [17,18]; however, mHealth in a CHD population has not been thoroughly investigated to date. The recently completed Heart Exercise and Remote Technologies (HEART) trial (*N* = 171) demonstrated that a simple text message and Internet intervention was effective in general as well as cost-effective for increasing leisure time physical activity and walking, but not for increasing maximal oxygen uptake in people with CHD at 6 months after randomisation [19,20]. Moreover, qualitative data indicated that the HEART intervention was well-received, had positive effects on participants’ physical activity levels and was not considered burdensome [21]. Whilst HEART demonstrated the feasibility and effectiveness of implementing a mobile telephone intervention, it focused on only a single behaviour (exercise) [22]. To build on this work, we propose testing the effectiveness of a comprehensive mHealth self-management intervention to enhance patients’ lifestyles.

Previous studies in which investigators targeted multiple risk factors often measured only one behaviour rather than the overall effect of the intervention [23]. Evaluating the combined effect of the intervention to change multiple behaviours is important, as many CHD patients have more than one lifestyle-related risk factor. The primary aim of our present trial (Text4Heart) is to evaluate the effectiveness of a comprehensive mHealth CR program in improving adherence to recommended lifestyle behaviour guidelines at 6 months after randomisation, in addition to usual CR care. Secondary objectives include the effect of 6 months of the intervention on overall cardiovascular (CVD) risk, body composition, illness perceptions, medication adherence, self-efficacy and hospital anxiety and/or depression.

## Methods/design

This protocol describes a six-month, two-arm, parallel, randomised controlled trial to evaluate an mHealth-delivered comprehensive (that is, multiple health behaviour) CR intervention in adults with CHD on the basis of a composite health behaviour score. The protocol is in accord with the SPIRIT 2013 statement [24], and the intervention is described according to the CONSORT-EHEALTH checklist [25]. See Additional file 1 for the complete checklist.

### Study sample and recruitment

Eligible participants are English-speaking New Zealand adults (ages 18 years and older) with a documented diagnosis of CHD (myocardial infarction (MI), angina or revascularisation) who meet the criteria for usual CR care and have access to the Internet (for example, library, work or home access). A basic mobile telephone with which to receive text messages will be available on loan to participants who do not own one (fewer than one in ten New Zealanders). Exclusion criteria are untreated ventricular tachycardia, severe heart failure, life-threatening coexisting disease with life expectancy less than 1 year, and/or significant exercise limitations for reasons other than CHD.

Potential participants will be screened during hospital admission at two metropolitan hospitals in Auckland, New Zealand. The annual incidence of hospitalisation for CHD in New Zealand overall is approximately 162 people per 100,000 [26] and over 2,400 in the Auckland area. The recruitment strategy was effective in the HEART trial [20,21]. Potential participants will receive a study information sheet while in hospital, and informed consent will be obtained from participants at the baseline assessment. All patients will be offered usual CR care. Assessments will be conducted in hospital, community clinics or participants’ homes to encourage retention.

### Outcome assessments

**Figure 1:**
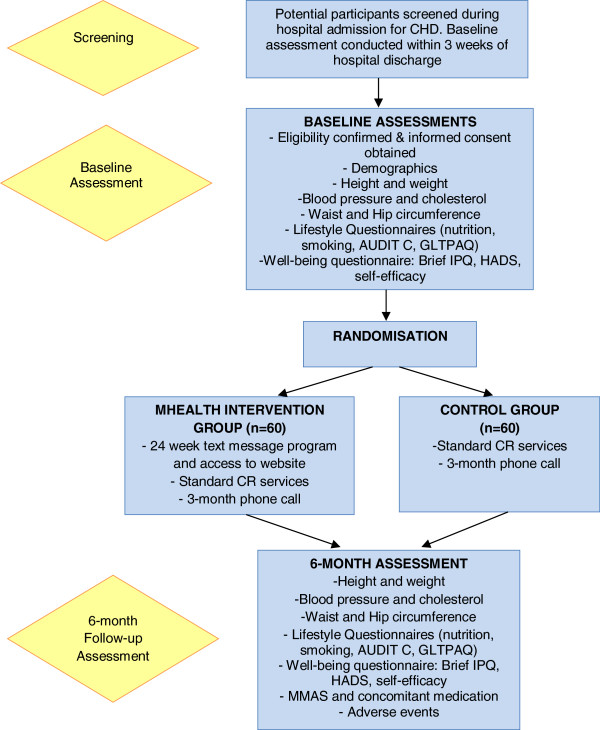
**Flow diagram of the study protocol.** AUDIT C: Alcohol Use Disorders Identification Test alcohol consumption questions; Brief IPQ: Brief Illness Perception Questionnaire; CHD: Coronary heart disease; CR: Cardiac rehabilitation; GLTPAQ: Godin Leisure Time Physical Activity Questionnaire; HADS: Hospital Anxiety and Depression Scale; MMAS: Morisky 8-item Medication Adherence Scale.

Assessments will be conducted at baseline and 6 months postrandomisation (see Figure 1). Baseline assessments will involve an explanation of study procedures, signed consent and collection of demographic details. Participant-reported primary and secondary outcomes will then be collected, followed by physical measurements, including height, weight, waist and hip circumference, blood pressure and blood cholesterol. The baseline assessment concludes with randomisation and assignment of participants to the respective study groups.

### Ethical approval

Ethical approval was obtained from the Health and Disability Ethics Committee (13/NTA/6). Each hospital research committee also approved the study. Serious adverse events will be collected at 12 weeks and 6 months and will be reported to the ethics committees along with any protocol amendments or violations.

### Sample size

One hundred twenty participants (sixty per group) will provide at least 80% power at the 5% level of significance (two-sided) to detect an absolute difference of 25% between the two groups, in the proportions of participants adherent to recommended healthy behaviour guidelines (that is, not smoking, low alcohol consumption, fruit and vegetable intake and engaging in physical activity) at 6 months after randomisation.

The sample size calculation was based on data from the EPIC-Norfolk Prospective Population Study, in which the investigators used a composite health behaviour score to determine the relationship between lifestyle and total mortality by cause in adults between 45 and 79 years of age living in the general community [27]. Given our population of people with established CHD, we estimate that 30% to 40% of our sample will be adherent to healthy behaviour guidelines at baseline [27].

### Randomisation and blinding

Following baseline data collection, eligible and consented participants will be randomised to either the intervention group or the control group in a 1:1 ratio. The randomisation sequence will be generated by using a computer program in blocks of six and will be overseen by the project statistician and unavailable to outcome assessors. Randomisation will be stratified according to smoking status (smoker vs. nonsmoker) to help balance baseline health behaviour scores. Allocation will be concealed in consecutively numbered, opaque sealed envelopes. Owing to the nature of the intervention, participants will be aware of the group to which they have been assigned; however, at follow-up, outcome assessors will be blinded to the treatment allocation.

### Standard cardiac rehabilitation care

All participants will be offered the standard outpatient CR program provided by each hospital, which involves education classes and supervised exercise.

### Study intervention

In addition to standard CR care, the mHealth intervention group will receive the core components of CR delivered via text messages (also known as *short messaging service* (SMS)) and a supporting website over the course of 24 weeks. The intervention will be delivered primarily by SMS because SMS is readily available to all mobile telephone users, inexpensive and easy to use. Participants will be offered brief training in how to use SMS and the Internet if necessary. Each intervention participant will receive a NZ$20 telephone credit voucher to reimburse any costs associated with replying to intervention messages. No changes will be made to the intervention or study design once recruitment begins; however, participants can choose to stop receiving the intervention and will be informed of this option during the consent process. A SMS library of 503 messages has been developed. It is written in English at an appropriate reading level (RMS 800 Lexile: approximately age 13 years) tested using the Lexile Analyzer 2013 software program (MetaMetrics, Durham, NC, USA). Intervention participants will receive five to seven messages per week for 24 weeks.

The overall goals of the intervention are to have individuals adhere to clinician-prescribed New Zealand CHD treatment [8] and American College of Sports Medicine guidelines [28]. Intervention content is based on the current CR guidelines, previous trials [19,29,30] and developmental studies. Content will be delivered on two aspects of self-management to improve participants’ condition and manage the emotional distress often associated with CHD: illness perceptions and lifestyle changes.

### Illness perceptions

Messages will address illness perceptions and medication-related beliefs using the Common-Sense Model [31]. The intervention will focus on modifying people’s perceptions of treatment for secondary prevention of CHD with regard to symptoms, timelines, causes, consequences, understanding of CHD treatment, personal control over CHD treatment and the effectiveness of CHD treatment. A lower perceived threat of illness has been shown to predict CR attendance and return to work in MI patients [32]. Messages will contain information and strategies to help participants stick to their prescribed medication regimen, information on the value of taking medication in terms of reducing subsequent events and hospitalisation, and reminders to have a regular checkup with their physicians.

### Lifestyle changes

The intervention includes education and support to eat a healthy diet, manage stress, exercise regularly, reduce alcohol consumption and stop smoking (if needed). Additional SMS will be delivered based on the suboptimal behaviour participants most wish to modify (outlined below), which will be identified at the baseline assessment.

#### Physical activity

Content was adapted from the HEART trial, and messages will be sent regarding the importance of being physically active, suggested activities and key strategies (for example, goal-setting and self-monitoring) to enhance uptake and maintenance of physical activity. General exercise prescription will be offered, detailing the type, frequency, duration and intensity of exercise based on participants’ preferred activities [28]. A pedometer will be provided to participants in the intervention group to assist with self-monitoring of daily activity.

#### Heart healthy diet

Participants will be supported to eat a heart-healthy diet and manage their weight. This support will include large servings of fruits and vegetables, whole grains, lean meats and alternatives while limiting intake of saturated fat, salt, alcohol and sugar [8]. Participants will receive text messages promoting healthy eating strategies, overcoming barriers and advice on choosing healthy food and food preparation.

#### Stress management

Participants will receive education to improve their quality of life through the identification and treatment of psychological distress. Treatment includes learning relaxation techniques and specific coping strategies to be used during times of stress, as well as avoiding harmful behaviours (that is, alcohol consumption). Strategies will be emphasised that facilitate participants’ return to a full and active life by enabling the development of their own resources. A health psychologist has aided in the development of these messages.

#### Stopping smoking

Participants who smoke tobacco will receive components of successful cessation interventions by text messaging [33] and video messaging [29]. They will be sent regular messages providing smoking cessation advice and support (for example, symptoms to expect upon quitting, tips to avoid weight gain during and after cessation and to cope with craving, and advice on avoiding smoking triggers).

The lifestyle change component is framed by social cognitive theory (SCT) [34]. A key construct of SCT is self-efficacy, or the confidence to engage in a desired behaviour, which has been shown to be a mediator of behaviour change [35] and is an outcome of CR [36]. Specific behaviour change techniques (BCTs) [37] that have been reported to be effective in changing health behaviours in a CHD population [38,39] will be used to target the constructs of SCT. All text messages are coded according to their SCT construct and related BCTs (see Additional file 2 for a sample).

### Supporting website

A secondary component of the intervention is a secure supporting website (Text4Heart homepage, https://text4heart.co.nz/, which is archived by WebCite at http://www.webcitation.org/6KNUFTSwm; see Additional file 3 for screenshots). A number of interactive features have been created to increase interactivity and website usage [30]. The website includes a blog whereby participants can ask questions and receive answers and social support, as well as a graph with which participants can monitor their physical activity by texting their pedometer counts, which will then be automatically uploaded. Additional information will be provided regarding CR, taking medications, various forms of physical activity, healthy eating, smoking cessation and hyperlinks to other websites (for example local exercise programs and cardiac clubs).

Efficacious beliefs can be enhanced through the use of vicarious learning, or *role modelling*, which refers to the process of learning behaviours by viewing other people’s behaviours and the outcomes of those behaviours [36]. Role modelling will be incorporated into the intervention through the use of short videotape messages (30 to 60 seconds) on the participant website. The video clips will involve a variety of CHD patients discussing their experiences of lifestyle change, the types of problems they faced, how they coped and any advice they can offer. Brief vignettes outlining the benefits of CR and healthy lifestyles, physiological responses, and safety issues will be provided by exercise scientists, nutritionists and cardiologists. Participants will receive occasional text messages (approximately once per fortnight) prompting them to view the website.

### Tailoring

A recent mHealth review found that personalised interventions were more effective at changing behaviour; however, few study researchers had implemented tailored components [17]. The Text4Heart intervention is personalised according to each participant’s name, choice of suboptimal behaviour and the time of day messages are sent. Bidirectional messages are included that require the participant to respond, such as texting in pedometer step counts, which then trigger a tailored response from the study team. Participants will be able to ‘text an expert’ to request personalised feedback, with questions answered by the researchers within 48 hours. This dynamic feedback loop holds promise to improve health behaviour, as rapid two-way communication provides participants with just-in-time information or strategies [40]. The intervention is designed to be automated and thus minimize human involvement; however, participants will be encouraged to connect with a real person (study team member) to ask questions if needed.

The corresponding author is responsible for monitoring the website and responding to incoming text messages from participants, as well as for updating the video page and blog with news and tips twice per week. The only planned personal contact from the study team will occur at 12 weeks after randomisation, when a member of the research team will telephone participants to remind them about the study, check on their progress against goals and answer any questions. Both the intervention and control groups will receive this telephone call to ensure that any between-group differences are the result of the text messages and the website, not human contact. The multifaceted approach of personalised SMS, videotape messages, website usage and opportunities for telephone contact may better encourage disease self-management than a unifaceted, SMS-only intervention [41].

### Primary outcome

The primary outcome is adherence to recommended lifestyle behaviours, which will be measured using a composite health behaviour score adapted from the EPIC-Norfolk Prospective Population Study [27]. In the present study, the measures described below will be used to determine participants’ health behaviour scores.

1. Smoking status will be measured using a common smoking history questionnaire [42]. This eight-item questionnaire is used to assess smoking status (current smoker, ex-smoker or nonsmoker), the number of cigarettes smoked per day and history of quit attempts.

2. Physical activity level will be assessed using the Godin Leisure Time Physical Activity Questionnaire (GLTPAQ) [43]. This simple three-item questionnaire has well-established reliability and validity and has been used in patients undergoing CR (*N* = 826) [44].

3. Alcohol consumption will be measured using the Alcohol Use Disorders Identification Test alcohol consumption questions (AUDIT-C) [45], a screening tool designed to assess units of alcohol consumed per week and to identify people who are hazardous drinkers. The AUDIT-C has been used as a primary care screening test for alcohol dependence in a sample of 243 older men with medical conditions, including heart disease [45]. Index cards referencing standard drink sizes will be used to reduce comprehension errors.

4. Fruit and vegetable intake will be assessed by two New Zealand–specific questions used in the 2006/2007 New Zealand Health Survey (*N* = 12,488, including adults with CHD) [46]. Index cards referencing standard fruit and vegetable serving sizes will be used to aid recall.

Participants will receive a score on a four-point scale, with one point each assigned for being a current nonsmoker, meeting physical activity guidelines to achieve some health benefits (14 units or more as scored on the GLTPAQ), consuming fourteen or fewer standard alcoholic drinks per week [27] and consuming at least five servings of fruits and vegetables per typical day [27].

### Secondary outcomes

The following are the secondary outcomes of the present study.

1. We will measure the difference in absolute CVD risk score [47] at 6 months after randomisation. This measure was developed for predicting risk in people with previous CVD events, but has also been applied to secondary prevention populations as a composite measure of changes in risk factors. The individual risk factors that form the absolute CVD risk score include age, lipid levels, blood pressure, and smoking and diabetes status.

2. Body composition will be measured using body mass index and waist-to-hip ratio. Physical measurements will be taken as the average of two readings.

3. Illness perceptions will be assessed using the Brief Illness Perception Questionnaire (B-IPQ) [48]. The B-IPQ has been used in a cardiac setting to examine speed of recovery following MI [32]. Items are scored on scales from zero to ten and can be collapsed into smaller clusters of causal beliefs. When items are combined, a higher score represents a more threatening perception of the participant’s disease.

4. Self-reported medication adherence will be assessed using the Morisky 8-item Medication Adherence Scale (MMAS) [49]. The MMAS has been used in a sample of patients with hypertension and has been found to predict reasons for poor adherence [50]. It will be completed at the 6-month assessment, as at baseline participants may not yet have been discharged from hospital with their new medications. Items are answered as yes or no, and scores are combined to indicate low, medium and high medication adherence. A list of participants’ prescribed medications will also be collected.

5. Self-efficacy will be assessed using the Self-efficacy for Managing Chronic Disease 6-item Questionnaire [51]. This six-item questionnaire has well-established reliability and validity on the basis of its use in a sample of participants with chronic disease, including heart disease (*N* = 1,130). Items are scored from one to ten, with a higher combined score indicating higher self-efficacy.

6. Mood will be assessed using the Hospital Anxiety and Depression Scale (HADS) [52]. The HADS performed well in assessing anxiety disorders and depression in somatic, psychiatric and primary care patients as well as in the general population. Participants will be asked to choose one of four answers to each item (six items for depression and six for anxiety). Each answer is assigned a score and added up to determine the indicated normal to abnormal levels of anxiety and/or depression.

7. Engagement in the intervention (for example, number of messages read, visits to the study website and number of contacts with the study team) will be assessed in exit surveys and on the basis of website usage statistics gathered by a website visit tracking system.

### Statistical analyses

Treatment evaluation will be performed on the principle of intention to treat. Statistical analyses will be conducted using SAS version 9.3 software (SAS Institute, Cary, NC, USA). The present study is a standard two-arm equality trial; therefore, all statistical tests will be two-sided and maintained at a 5% significance level. Demographics and baseline characteristics of all randomised participants will be summarized for each group as well as overall. Continuous variables will be reported as mean ± SD. Categorical variables will be reported as numbers (%).

Simple χ^2^ analyses will be used to evaluate the main treatment effect on the proportion of participants adherent to health behaviours at end of the intervention period, with estimation of relative risks, 95% confidence intervals and two-sided *P*-values. We hypothesise that the proposed intervention will change the proportion of participants adherent to recommended healthy behaviour guidelines at 6 months after randomisation by at least 25% compared with the control group. Multiple regression analysis appropriate for binary and continuous outcome measures will be conducted to evaluate the treatment effects, adjusting for important confounding factors at baseline. All analyses for secondary outcomes will be exploratory.

## Discussion

The Text4Heart trial will evaluate a comprehensive mHealth CR intervention to improve adherence to lifestyle behaviours. To the best of our knowledge, this is the first mHealth study to assess multiple behaviours using a composite score and one of the first to evaluate a comprehensive mHealth self-management intervention in adults with CHD. The protocol, in accordance with the SPIRIT statement, incorporates findings from recent mHealth systematic reviews with the aim of adding quality evidence to the body of literature.

Common criticisms have been voiced regarding mHealth research designs and trial descriptions [41,53]. Few adequately powered randomised controlled trials have been completed to date. Intervention duration and follow-up assessments are often not long enough, as it takes approximately 6 months for participants to adopt new behaviours [54]. The Text4Heart trial has been developed with these concerns in mind, and therefore we have incorporated a randomised controlled trial design with a 6-month follow-up period.

Many mHealth studies also fail to fully describe their interventions, in particular the behaviour change theory or BCTs used, if any, and the evidence behind the content of the intervention [25,53]. mHealth studies often offer insufficient explanation of the intervention for its design to be replicated [25]. This is a particular concern because mHealth technology changes rapidly, and it is important to know which aspects of an intervention worked and which did not. The Text4Heart study protocol describes the intervention in full, including the theoretical framework and examples of SMS and Internet content. The intervention incorporates knowledge learned from previous mHealth research and has involved the target audience in the content development stage of the intervention.

On the basis of earlier results, it is evident that the CHD population in New Zealand is interested in mHealth CR and is able to use the related technology [20,55]. If effective, the trial has the potential to be implemented in a CR setting and to be used as a model for other disease prevention and self-management programs. The intervention could easily be augmented to provide opportunities for clinicians to engage in two-way communication with patients regarding their lifestyle behaviours, as well as clinical outcomes such as monitoring blood pressure and weight. This study will provide data on the potential of mHealth to improve patient care.

## Trial status

The trial is currently open for recruitment.

## Abbreviations

BCT: Behaviour change technique; BIPQ: Brief Illness Perception Questionnaire; CHD: Coronary heart disease; CR: Cardiac rehabilitation; CVD: Cardiovascular disease; GLTPAQ: Godin Leisure Time Physical Activity Questionnaire; MMAS: Morisky 8-item Medication Adherence Scale; HADS: Hospital Anxiety and Depression Scale; SCT: Social cognitive theory; SMS: Short messaging service.

## Competing interests

The authors declare that they have no competing interests.

## Authors’ contributions

All authors contributed to the study concept, design and procedures. LP drafted the manuscript. JY created the statistical analysis plan and carried out the randomisation sequence. LPD, RM, RW, RS and AR contributed to the intervention content. Access to data is limited to LP, RM and YJ unless requested by others. All authors read and approved the final manuscript.

## Supplementary Material

Additional file 1 **SPIRIT 2013 Checklist.** The SPIRIT checklist lists items to be included in the protocol. All items are accounted for either in the manuscript or in this file. Click here for file

Additional file 2 **Example of text message content and related social cognitive theory (SCT) construct and behaviour change techniques (BCTs).** This table provides examples of intervention content and the related theoretical constructs. Click here for file

Additional file 3 **Text4Heart study website screenshots.** This document provides screenshots of the Text4Heart participant website. Click here for file
