# Supplementary material for: Improving coronary heart disease self-management using mobile technologies (Text4Heart): a randomised controlled trial protocol
Source: Trials. 2014 Mar 4;15:71. doi: 10.1186/1745-6215-15-71 (PMC4015816; doi:10.1186/1745-6215-15-71)
Supplement: Additional file 1 — SPIRIT 2013 Checklist. The SPIRIT checklist lists items to be included in the protocol. All items are accounted for either in the manuscript or in this file. [file 1745-6215-15-71-S1.docx]

Additional file 1. SPIRIT 2013 Checklist

| Section/Item | Item Number | Included in manuscript (Y/N) or described below |
| --- | --- | --- |
| **Administrative Information** |  |  |
| Title | 1 | Y |
| Trial registration | 2a | Y |
|  | 2b | Y |
| Protocol version | 3 | Version 3, 03/10/2013 |
| Funding | 4 | Y |
| Roles and responsibilities | 5a | Y |
|  | 5b | Y |
|  | 5c | Y |
|  | 5d | The steering committee is comprised of the study authors. The National Institute for Health Innovation’s IT team are responsible for the SMS delivery and website design and function. |
| **Introduction** |  |  |
| Background and rationale | 6a | Y |
|  | 6b | Y |
| Objectives | 7 | Y |
| Trial design | 8 | Y |
| **Methods** |  |  |
| Participants, interventions, and outcomes | | |
| Study setting | 9 | Y |
| Eligibility criteria | 10 | Y |
| Interventions* | 11a | Y |
|  | 11b | Y |
|  | 11c | Y |
|  | 11d | Y |
| Outcomes | 12 | Y |
| Participant timeline | 13 | Y |
| Sample size | 14 | Y |
| Recruitment | 15 | Y |
| Assignment of interventions |  |  |
| Allocation |  |  |
| Sequence generation | 16a | Y |
| Allocation concealment mechanism | 16b | Y |
| Implementation | 16c | Y |
| Blinding (masking) | 17a | Y |
|  | 17b | The PI will not be blinded as she registers intervention participants into the text message delivery system. Outcome assessors will be blinded, and it will not be necessary to unblind them. |
| Data collection, management and analysis | | |
| Data collection methods | 18a | Y – Data collection forms found in the trial master file |
|  | 18b | Y |
| Data management | 19 | Y - Data will be entered by outcome assessors into a password protected secure Microsoft Excel spread-sheet. Data range checks will be in place to promote data quality. Data will be manually checked against source documents periodically throughout the study by a monitor external to the study. The trial will be subjected to independent auditing. |
| Statistical methods | 20a | Y |
|  | 20b | N/A – no subgroup analyses are planned |
|  | 20c | Y |
| Monitoring |  |  |
| Data monitoring | 21a | Y - Data monitoring forms available from the trial master file upon request |
|  | 21b | N/A: No interim analyses are planned; the trial is low risk |
| Harms | 22 | Y |
| Auditing | 23 | Y |
| **Ethics and dissemination** |  |  |
| Research ethics approval | 24 | Y |
| Protocol amendments | 25 | Y – amendments will be communicated to steering committee, ethics boards, and trial registry if needed |
| Consent or assent | 26a | Y |
|  | 26b | Y – information on blood cholesterol test included in consent form |
| Confidentiality | 27 | Y – all participants are assigned a unique registration number used on data collection forms |
| Declaration of interests | 28 | Y |
| Access to data | 29 | Y |
| Ancillary and post-trial care | 30 | Y – described in consent form |
| Dissemination policy | 31a | Y |
|  | 31b | Y – no professional writers will be used |
|  | 31c | Y – there are no plans to make data set public |
| **Appendices** |  |  |
| Informed consent materials | 32 | Y – Version 2, 02/10/2013, found in trial master file |
| Biological specimens | 33 | Y – described in consent form |

*Additional information has been included in the manuscript to follow the CONSORT-EHEALTH item 5 checklist [26]. All sub-items in the checklist have been described.
