# Supplementary material for: Improving coronary heart disease self-management using mobile technologies (Text4Heart): a randomised controlled trial protocol
Source: Trials. 2014 Mar 4;15:71. doi: 10.1186/1745-6215-15-71 (PMC4015816; doi:10.1186/1745-6215-15-71)
Supplement: Additional file 2 — Example of text message content and related social cognitive theory (SCT) construct and behaviour change techniques (BCTs). This table provides examples of intervention content and the related theoretical constructs. [file 1745-6215-15-71-S2.docx]

Additional file 2. Example of text message content and related SCT construct and BCT

| SCT construct [35] | BCT [38] | Example |
| --- | --- | --- |
| *Knowledge* | 1: *Provide information about behaviour-health link* | T4H: High cholesterol or high blood pressure is not good for your heart condition. Your mediations will help improve these |
|  | 8: *Provide instruction* | T4H: Nutrition labels on your packaged food tell you how much fat & salt your food contains per 100g. Compare products & pick the lowest |
| *Outcome expectations* | 2: *Provide information on consequences* | T4H: Lower your risk of future heart problems by making healthy changes. Take your pills, keep active, and get your 5 fruits & veg each day |
| *Perceived self-efficacy*, created through 4 sources: |  |  |
| *Mastery experiences* | 7: *Set graded tasks* | T4H: It's week 3. Time to increase the length of your walk. Add a few more minutes this week. Warmup, walk, cool down |
| *Vicarious learning* | 9: *Model or demonstrate the behaviour* | Video role model messages |
|  | 19: *Provide opportunities for social comparison* | Video role model messages |
| *Social persuasion* | 6: *Provide general encouragement* | T4H: Well done, you have been going for 7 weeks now! Reward yourself with some ME time - try a favourite activity |
| *Interpretation of physiological states* | 24: *Stress management* | T4H: If you feel overwhelmed or stressed, try to set some time aside each day to relax. Close your eyes and do 5 minutes of deep breathing |
|  | Author-created BCT: *Interpretation and normalising of physical or emotional symptoms* when changing behaviour (i.e.: heart should beat faster when exercising) | T4H: It's normal to huff & puff during exercise. If this happens at rest or continues after exercise, this is not normal and you should see your GP |
| *Self-regulation* | 4: *Prompt intention formation* | T4H: The key to goal setting is to set small goals, make them stick by writing them down, and reading them regularly |
|  | 5: *Prompt barrier identification* | T4H: Too tired after work to cook a healthy dinner? Try getting up a bit earlier and prepare it in the morning or cook on the weekend and freeze |
|  | 10: *Prompt specific goal setting* | T4H: Add a new goal - how about a competition? Challenge someone at home or via the online blog to do better than you this week |
|  | 11: *Prompt review of behavioural goals* | T4H: You're a few weeks into the program. Well done! Time to add a new goal - maybe you are ready to think about a long term goal this time |
|  | 12: *Prompt self-monitoring of behaviour* | T4H: Hi [name], time to text me your step count (reply with a number) and I'll post it online. Thanks! |
|  | 13: *Provide feedback on performance* | T4H: Hi [name], thanks 4 your text. You're on your way to an active lifestyle. Think of how you can fit extra steps into your day. Every bit helps! |
|  | 20: *Plan social support* | T4H: If you are finding it hard to get people to support you, ask others in this study to help - blog it. Many will be feeling the same thing |
|  | 26: *Time management* | T4H: Some people like to exercise in the morning and some in the afternoon. Pick a time that suits you and make exercise a habit |
