# Supplementary figures and images for: Improving coronary heart disease self-management using mobile technologies (Text4Heart): a randomised controlled trial protocol
Source: Trials. 2014 Mar 4;15:71. doi: 10.1186/1745-6215-15-71 (PMC4015816; doi:10.1186/1745-6215-15-71)

Additional file 3. Text4Heart study website screenshots


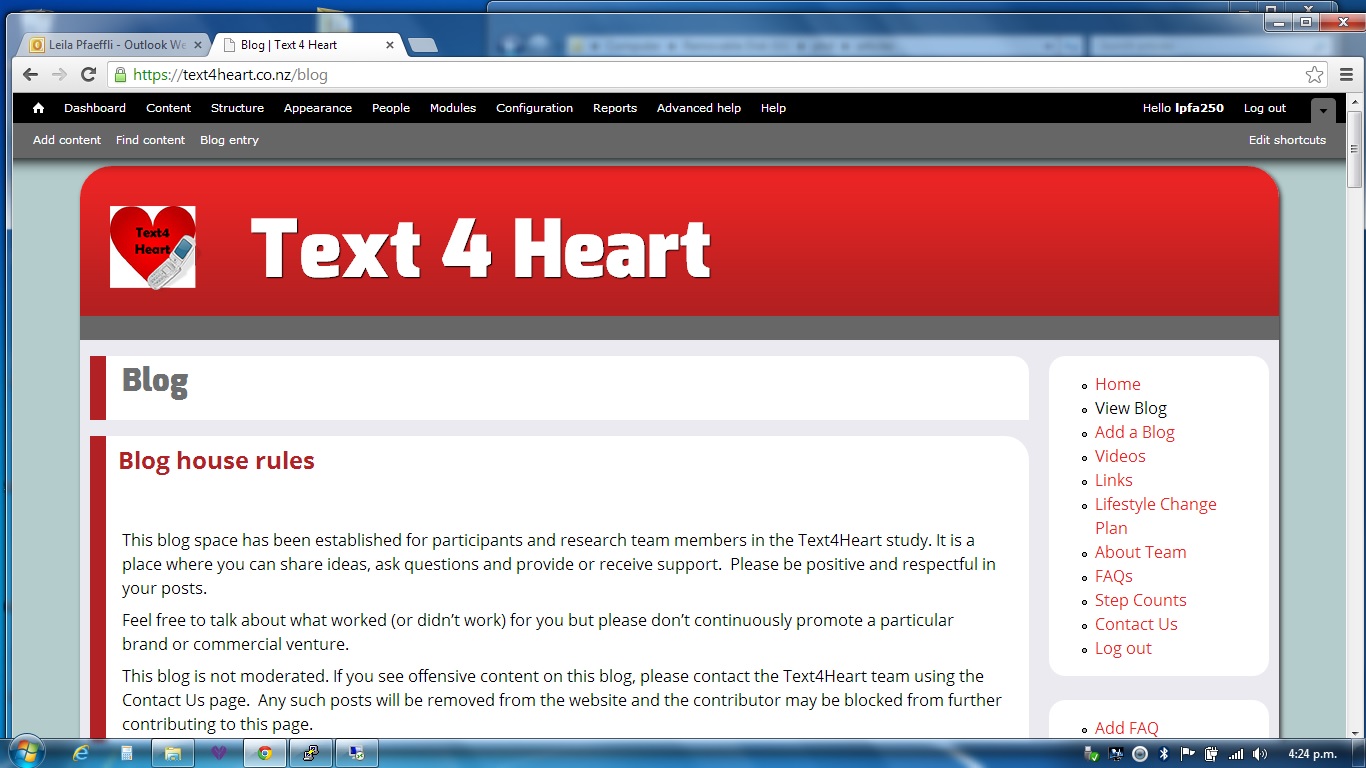


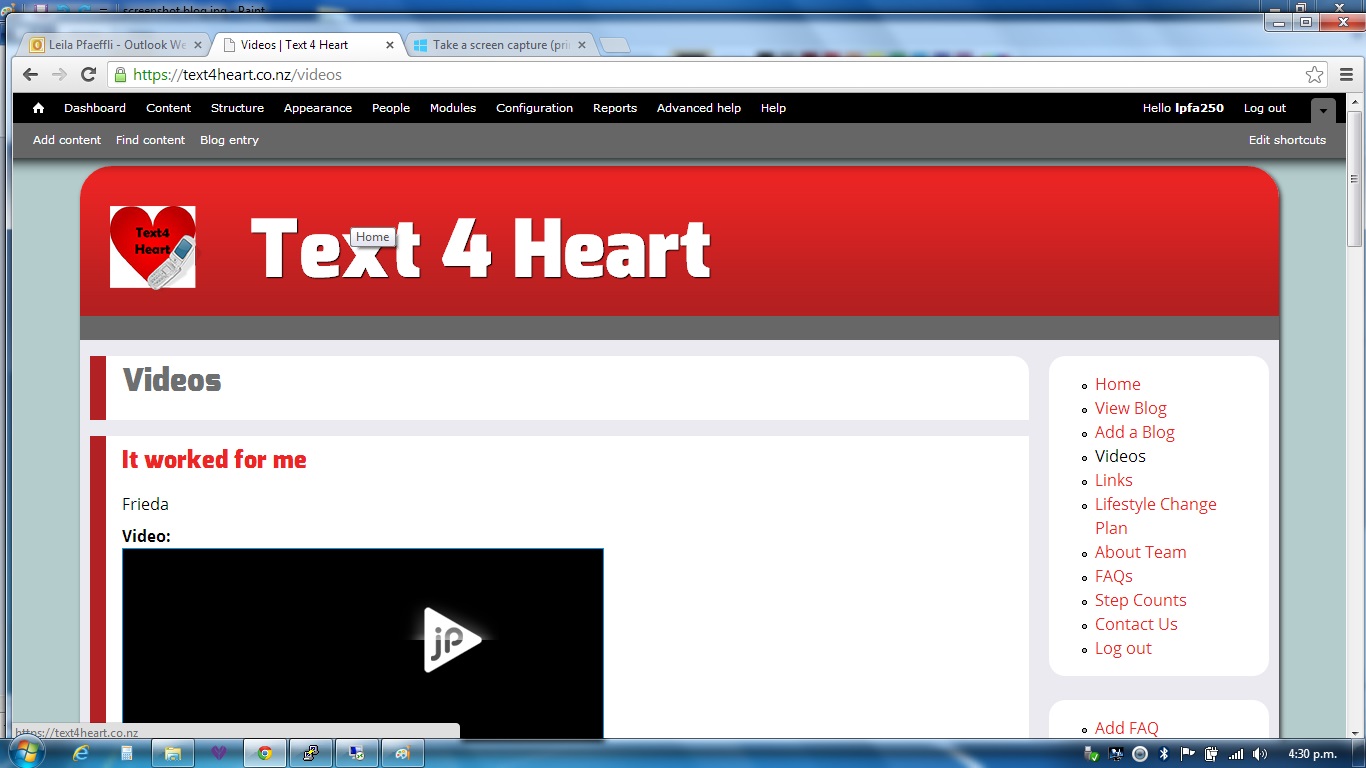


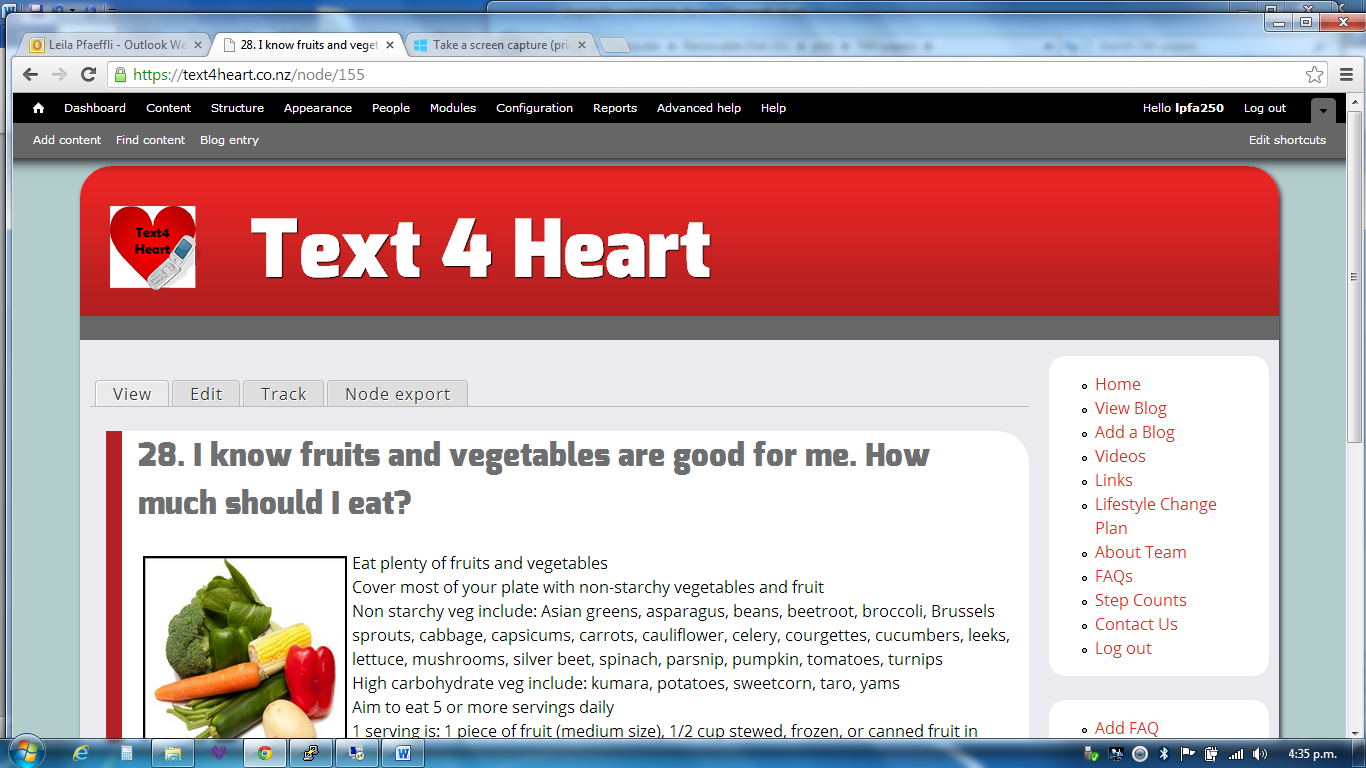

Supplement: Additional file 3 — Text4Heart study website screenshots. This document provides screenshots of the Text4Heart participant website. [file 1745-6215-15-71-S3.docx]
